# Polyketides, Toxins and Pigments in *Penicillium marneffei*

**DOI:** 10.3390/toxins7114421

**Published:** 2015-10-30

**Authors:** Emily W. T. Tam, Chi-Ching Tsang, Susanna K. P. Lau, Patrick C. Y. Woo

**Affiliations:** 1 Department of Microbiology, The University of Hong Kong, Pokfulam, Hong Kong; E-Mails: emily.wt@gmail.com (E.W.T.T.); microbioct@connect.hku.hk (C.-C.T.); 2 State Key Laboratory of Emerging Infectious Diseases, The University of Hong Kong, Pokfulam, Hong Kong; 3 Research Centre of Infection and Immunology, The University of Hong Kong, Pokfulam, Hong Kong; 4 Carol Yu Centre for Infection, The University of Hong Kong, Pokfulam, Hong Kong

**Keywords:** *Penicillium marneffei*, polyketide synthase, pigment

## Abstract

*Penicillium marneffei* (synonym: *Talaromyces marneffei*) is the most important pathogenic thermally dimorphic fungus in China and Southeastern Asia. The HIV/AIDS pandemic, particularly in China and other Southeast Asian countries, has led to the emergence of *P. marneffei* infection as an important AIDS-defining condition. Recently, we published the genome sequence of *P. marneffei*. In the *P. marneffei* genome, 23 polyketide synthase genes and two polyketide synthase-non-ribosomal peptide synthase hybrid genes were identified. This number is much higher than those of *Coccidioides immitis* and *Histoplasma capsulatum*, important pathogenic thermally dimorphic fungi in the Western world. Phylogenetically, these polyketide synthase genes were distributed evenly with their counterparts found in *Aspergillus* species and other fungi, suggesting that polyketide synthases in *P. marneffei* did not diverge from lineage-specific gene duplication through a recent expansion. Gene knockdown experiments and ultra-high performance liquid chromatography-photodiode array detector/electrospray ionization-quadruple time of flight-mass spectrometry analysis confirmed that at least four of the polyketide synthase genes were involved in the biosynthesis of various pigments in *P. marneffei*, including melanin, mitorubrinic acid, mitorubrinol, monascorubrin, rubropunctatin, citrinin and ankaflavin, some of which were mycotoxins and virulence factors of the fungus.

## 1. Introduction

*Penicillium marneffei* (synonym: *Talaromyces marneffei*) is one of several known thermally dimorphic fungi and an important pathogen endemic in tropical Southeast Asian countries. *P. marneffei* causes respiratory, cutaneous and subcutaneous, systemic, and even disseminated mycoses in humans [1,2,3,4]. Since *P. marneffei* was first discovered in 1956, there have only been 18 reported human cases of penicilliosis until 1985 [5]. The emergence of the HIV/AIDS pandemic in the 1980s, especially in the tropical Southeast Asian region, including China, has led to a surge of opportunistic mycotic infections in HIV-positive, immunocompromised patients, which were often caused by *P. marneffei*. Hong Kong, situated in the tropical Southeast Asian area, is also affected by *P. marneffei* infections and approximately 8% of patients with HIV/AIDS in this city are infected with this pathogenic fungus [6]. Moreover, in the northern part of Thailand, *P. marneffei* infection, following tuberculosis and cryptococcosis, is the third commonest indicator disease of HIV/AIDS [2]. Apart from HIV-positive patients, other immunocompromised patients are also susceptible to penicilliosis [7,8,9,10], and *P. marneffei* infections are found in patients with autoantibody against interferon-γ with an increasing trend [11,12,13]. Recently, we have described the emergence of *P. marneffei* infections in hematological patients receiving targeted therapies [14].

Polyketides are a broad class of secondary metabolites synthesized by microbial organisms. Although not essential, these secondary metabolites are biologically active and could provide survival advantages to the microbial hosts. Some well-known secondary metabolites produced by fungi include antibiotics (e.g., cephalosporin, myriocin and penicillin), mycotoxins (e.g., aflatoxin, fumonisin, ochratoxin and zearalenone), and pigments (e.g., aurofusarin, bikaverin and melanin). As for *P. marneffei*, a variety of pigments are produced by this fungus, and the pigment composition varies with the fungal cell types and morphological forms. For example, young conidia of *P. marneffei* appear black in color, and they turn yellow upon maturation. On the other hand, when cultured as the yeast form, the colonies of *P. marneffei* are creamy in color. In addition, *P. marneffei* in mycelial form is well known to secrete a diffusible red pigment. This is an important diagnostic property of this fungus in clinical microbiology laboratories.

Polyketides are synthesized via a complex enzymatic system consisting of various kinds of polyketide synthases (PKSs). Genes that encode these PKSs (*pks* genes) are located in close proximity to each other. Close to these *pks* gene loci there are additional genes encoding modifying enzymes, and the *pks* genes form biosynthetic gene clusters together with these modifying enzyme genes. The availability of more and more microbial genomes has allowed us to predict various biological properties of microorganisms and the corresponding biosynthetic genes, gene clusters and even pathways. For *P. marneffei*, based on its draft genome sequence, the mitochondrial genome and genes related to its predicted sexual cycle have been analyzed [15,16]. A highly discriminative multi-locus sequence typing scheme was also developed [17]. It was found that the genome of *P. marneffei* contains a total of 25 putative *pks* genes [18], which is much higher in number than in other thermally dimorphic fungi, where there are only one *pks* gene for *Histoplasma capsulatum* and ten for *Coccidioides immitis* [19]. It is common that the species of the phylum Ascomycota have a large number of *pks* genes. Since *P. marneffei* is the only thermally dimorphic fungus belonging to Ascomycota, it may explain why *P. marneffei* also possesses a large number of PKS genes. In this article, the diversity and phylogeny of these *pks* genes in *P. marneffei*, the *pks* genes responsible for the biosynthesis of the various pigments, as well as the biological properties and biosynthetic pathways of these pigments are reviewed.

## 2. Diversity and Phylogeny of *pks* Genes in *P. marneffei*

**Figure 1:**
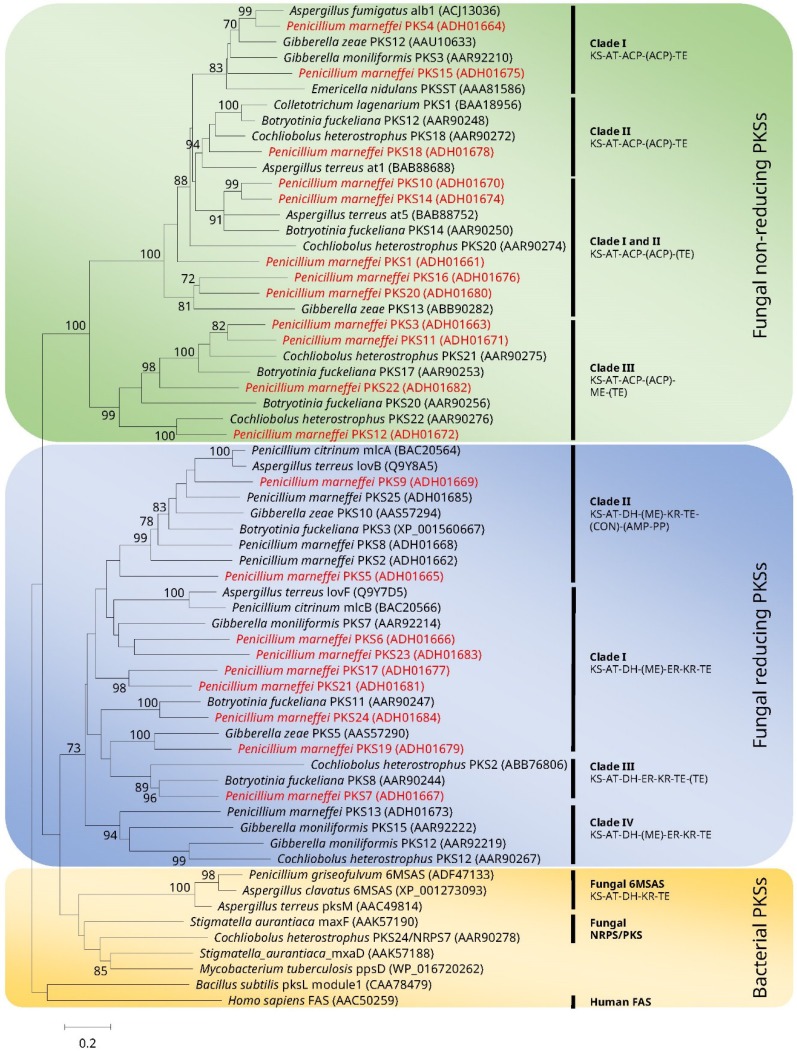
Phylogenetic tree showing the relationship of the polyketide synthases (PKSs) of *Penicillium marneffei* with other organisms, inferred from the partial ketosynthase domain amino acid sequence data by the maximum likelihood method using the substitution model WAG (Whelan and Goldman model) + F (estimated of amino acid frequency) + G (gamma-distributed rate variation) + I (estimated proportion of invariable sites). The scale bar indicates the estimated number of substitutions per amino acid residue. All names and accession numbers are given as cited in the DDBJ/ENA/GenBank databases. Numbers at nodes indicate levels of bootstrap support calculated from 1 000 trees and are expressed as percentage. Only nodes that were well supported by the maximum likelihood method (≥70% bootstrap support) have their bootstrap values shown. Clades and the typical domain organization defined by Kroken *et al.* [22] are indicated. The 21 putative PKSs of *P. marneffei* which contained all the KS, AT, and ACP domains were highlighted in red color. A. adenylation; ACP, acyl carrier protein; AT, acyltransferase; C, condensation; DH, dehydrogenase; ER, enylreductase; KR, ketoreductase; KS, ketosynthase; MT, methyltransferase; T, thiolation; TE, thioesterase; and R, reductase. Adapted with permission from “Phylogenetic analysis of PKSs of *Penicillium marneffei*” in “Characterization of polyketide synthases in *Penicillium marneffei*” by W. T. Tam, 2012, PhD thesis submitted to the University of Hong Kong, p.93. Copyright 2012 licensed under Creative Commons: Attribution 3.0 Hong Kong License (https://creativecommons.org/licenses/by/3.0/hk/).

A total of 25 *pks* genes have been identified in the draft genome of *P. marneffei* [18]. For those fungi with available genome sequences, there are only: one *pks* gene in *H. capsulatum*, ten in *C. immitis*, 14 in *Aspergillus fumigatus*, 16 in *Gibberalla zeae*, 20 in *P. chrysogenum*, 27 in *A. nidulans*, and 30 in *A. oryzae* [19]. Although the diversity of *pks* genes in the genome of *P. marneffei* is similar to phylogenetically closely related fungi, such as *Aspergillus* species, the *pks* genes of *P. marneffei* are much more diverse than those of other thermally dimorphic fungi. This implies that *P. marneffei* could potentially produce a larger variety of polyketide metabolites than other clinically important thermally dimorphic fungi [18]. When the gene sequences of the PKS domains of *Aspergillus* spp. were used to BLAST against the *P. marneffei* genome in the GenBank database, it was found that 23 putative *pks* genes and two putative PKS-non-ribosomal peptide synthase (NRPS) hybrid genes (*pks-nrps2* and *pks-nrps8*) are present in the genome of *P. marneffei* [18,20,21]. Among the 23 gene candidates, 21 putative *pks* genes encode gene products with the ketosynthase (KS), acyltransferase (AT) and acyl carrier protein (ACP) domains, which are the constitutional components of PKS. The ACP domain is absent in the predicted gene products of the remaining two candidates (*pks13* and *pks25*), implying that these two genes might be pseudogenes [18]. The 21 putative *pks* genes with the KS, AT and ACP domains are clustered in 18 groups, where three of these groups contain two *pks* genes each (*pks11* and *pks12*, *pks16* and *pks17*, and *pks20* and *pks21*). These 18 groups of putative *pks* genes could potentially produce 18 different polyketide metabolites. Twelve of these 21 gene candidates belong to the non-reducing type, whereas the remaining nine belong to the reducing type, which, on top of the KS, AT and ACP domains, also contain the dehydrogenase (DH) and ketoreductase (KR) domains [18] (Figure 1). Eight of the nine *pks* genes belonging to the reducing type possess the enoylreductase (ER) domain. For the three gene clusters in the genome which contain two *pks* genes each, two clusters (*pks16* and *pks17*, and *pks20* and *pks21*) encode one non-reducing and one reducing PKS, and the third cluster (*pks11* and *pks12*) encodes two reducing PKSs. As for the two *pks-nrps* hybrid candidate genes (*pks-nrps2* and *pks-nrps8*), the PKS modules belong to the reducing type with DH and KR domains, and the whole NRPS modules possess the condensation (C), adenylation (A), thiolation (T) and thiolester reductase (R) domains [18].

Phylogenetic analysis of the KS domains revealed that PKSs in *P. marneffei* are scattered evenly in the phylogenetic tree with other fungal PKSs (Figure 1). This indicates that PKSs in *P. marneffei* did not diverge from lineage-specific gene duplication through a recent expansion. Moreover, the majority of the PKSs are grouped into the eight clades of the typical PKS phylogeny described by Kroken *et al.*, where fungal PKSs, bacterial PKSs and human fatty acid synthase (FAS) were included in the analysis [22] (Figure 1). The PKSs of *P. marneffei* in each of the clades also possess the same domain structures as other members of the corresponding clade. The 12 non-reducing PKSs of *P. marneffei* are clustered with the non-reducing PKSs of other fungi in the phylogenetic tree with high bootstrap support. Similarly, the nine reducing PKSs of *P. marneffei* are also clustered with the reducing PKSs of other fungi in the tree with high bootstrap support (Figure 1). This suggests that the non-reducing PKSs and reducing PKSs of *P. marneffei* share sequence and functional homologies with their respective counterparts found in other fungi.

## 3. Pigments and Toxins in *P. marneffei*

*P. marneffei* produces black and yellow pigments on its conidia. It also produces a diffusible red pigment, which readily diffuses to the surrounding medium. Studies over the past few years have revealed that some of the compounds which constitute these pigments represent known virulence factors for other fungi or novel virulence factors [18,21,23,24,25], whereas others represent pigments that have been used in various industries for over a thousand years [20,26,27,28].

### 3.1. Melanin

Melanin, a group of pigments usually in dark brown or black color, consists of negatively charged and hydrophobic compounds with high molecular weights. A wide range of organisms, including bacteria, fungi, plants and animals, produces melanin. In the 1960s, melanins were proven to exist in fungi. Many fungi that are pathogenic to human, such as *A. fumigatus* [29] and *Cladosporium carionii* [30], as well as all the known pathogenic thermally dimorphic fungi [24,31,32,33,34], produce melanin. The correlation of melanin to fungal pathogenesis has raised research interest in the scientific community.

In 2005, Youngchim *et al.* firstly reported the presence of melanin or melanin-like compounds in *P. marneffei* [35]. Using electron spin resonance (ESR) spectroscopy and detection of antibody response in mice model using melanin-binding antibody labeling, it was shown that *P. marneffei* produces melanin/melanin-like compounds *in vitro* and during infection in both the conidia and yeast cells [35]. In 2010, the dihydroxynaphthalene (DHN)-melanin biosynthetic gene cluster of *P. marneffei* was reported [18]. The cluster contains six genes, which encode two oxidases (ABR1 and ARB2), a pigment biosynthesis protein (AYG1), a scytalone dehydratase (ARP1), a 1,3,6,8-tetrahydroxynaphtalene reductase and a polyketide synthase (*pks4*, also named as *alb1*), and span over a 25.3 kb region of the fungal genome. This indicates that the fungus produces melanin via a PKS pathway [33]. The protein ALB1 is a non-reducing PKS and is composed of the KS-AT-ACP-ACP-thioesterase (TE) domains [18]. Compared with its homologs in other fungi, the domain organization of ALB1 is highly conserved [18]. The gene orientation and gene order in the cluster are the same as those found in the genome of *T. stipitatus*, a non-pathogenic saprophytic fungus which can be found in decaying plant materials and soil. Furthermore, all the six DHN-melanin-related genes in *P. marneffei* can also be found in other *Penicillium* species and *Aspergillus* species that are phylogenetically closely related to *P. marneffei* [36,37]. This indicates that the melanin biosynthesis gene cluster in *P. marneffei* has evolved in parallel with the evolution of the fungus. The melanin biosynthetic gene cluster was probably inherited from the common ancestor of this clade of fungi. Subsequent gene rearrangement and gene divergence events have led to different gene orientations and orders of the corresponding genes in different fungi. Recognized human pathogenic fungi which synthesize DHN-melanins include *A. nidulans*, *A. niger*, *Alternaria alternata*, *C. carionii*, *Exophiala jeanselmei*, *Fonsecaea compacta*, *F. pedrosoi*, *Hendersonula toruloidii*, *Phaeoannellomyces wernickii*, *Phialophora richardsiae*, *P. verucosa*, *Sporothrix schenckii*, *Wangiella dermatitidis* and *Xylohypha bantiana* [31,38,39,40].

It has been demonstrated that *Colletotrichum lagenarium* synthesizes DHN-melanin using malonyl-CoA as the starter and extender units using the polyketide synthase PKS1, which catalyzes the first step of melanin biosynthesis [41,42]. The first detectable intermediate of the melanin biosynthetic pathway is 1,3,6,8-tetrahydroxynaphthalene (1,3,6,8-THN), which is enzymatically reduced to scytalone by a reductase. Scytalone is then dehydrated to 1,3,8-trihydroxynaphthalene and, after a second reduction step, is converted to vermelone. A further dehydration step leads to the formation of the intermediate 1,8-DHN. Melanin is finally synthesized by subsequent dimerization and polymerization of the 1,8-DHN molecules, which are catalyzed by a laccase [43]. The DHN-melanin biosynthesis in *A*. *fumigatus* is similar to that in *P. marneffei*. ALB1, also known as PKSP, produces heptaketide naphtopyrone, which is converted to 1,3,6,8-THN by AYG1. By repetitive steps of reduction (catalyzed by ARP2) and dehydration (catalyzed by ARP1, a scytalone dehydratase; and ABR1, a multicopper oxidase), 1,3,6,8-THN is converted to 1,8-DHN, and is finally polymerized to DHN-melanin (catalyzed by ABR2, a laccase) [29,37]. Since the DHN-melanin biosynthetic cluster in *P. marneffei* also contains all these six genes (*abr1*, *arb2*, *ayg1*, *arp1*, *alb1*, and *arp2*), and all these genes are phylogentically closely related to the *A. fumigatus* counterparts, the biosynthesis of DHN-melanin in *P. marneffei* is believed to be similar to *A. fumigatus*.

**Figure 2:**
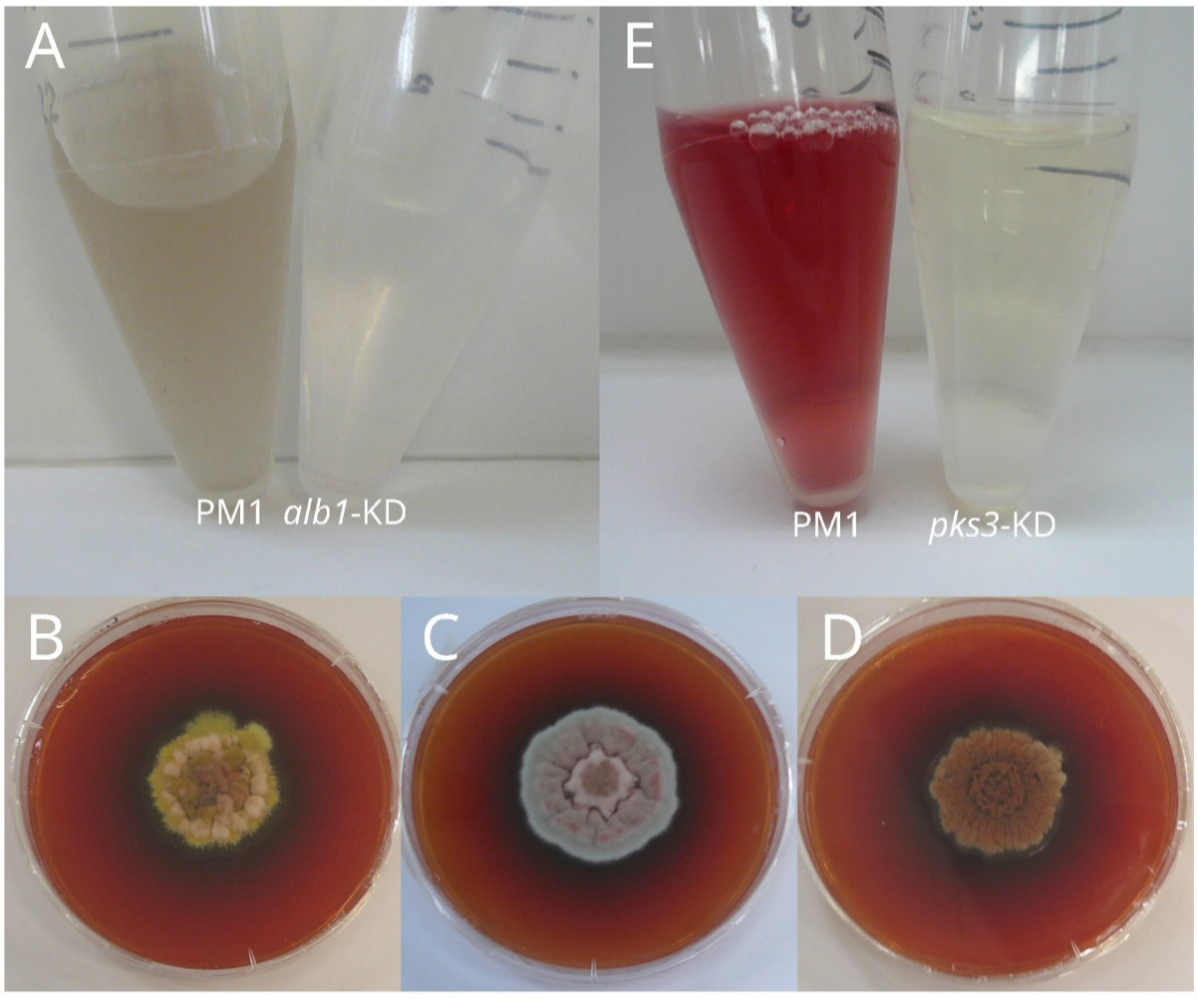
(**A**) Conidia suspensions of *Penicillium marneffei* wild type strain PM1 (**left**) and *alb1*-knockdown mutant (**right**). A loss of black pigment was observed in the conidia of the *alb1*-knockdown mutant; (**B**) Colony morphology of *P. marneffei* wild type strain PM1; (**C**) *pks11*-knockdown mutant; and (**D**) *pks12*-knockdown mutant on Sabouraud dextrose agar after 14 days of incubation at 25 °C. A loss of yellow pigment was observed in the conidia of the *pks11*- and *pks12-*knowckdown mutants; (**E**) Culture supernatants of *P. marneffei* wild type strain PM1 (**left**) and *pks3-*knockdown mutant (**right**) after 4 days of incubation in Sabouraud dextrose broth at 25 °C. A loss of red pigment production was observed for the *pks3*-knockdown mutant.

When *alb1* was knocked down (KD), the isogenic *P. marneffei* mutant showed a loss of melanin in its conidia (Figure 2A), a reduction in the degree of ornamentation on the conidia surface, an attenuation of virulence in mouse model and a decrease in resistance to hydrogen peroxide killing [18]. The melanin biosynthetic gene cluster may contribute to virulence by reducing the fungal susceptibility to hydrogen peroxide killing [18]. In other pathogenic fungi, including *A. fumigatus*, *P. brasiliensis* and *S. schenckii*, loss of melanin production would decrease the susceptibility of the fungi to hydrogen peroxide killing, the capability to hide pathogenic substances, as well as the induction of cytokine response [25,31,36,44]. Hence, the loss of the pigment melanin may result in a reduction in fungal virulence. Some non-pathogenic fungi, such as *T. stipitatus*, also bear the melanin biosynthetic gene cluster. This likely suggests that resistance against hydrogen peroxide killing is only one of the steps that gives rise to its pathogenic property [18].

Scanning electron microscopic examination showed that the degree of ornamentation on the conidia surface of *P. marneffei* melanin-KD mutant was lowered [18]. This observation was similar to that observed in *A. fumigatus*, where deletion of the *alb1* gene showed a loss of the outermost electron dense layer of its conidial surface [29]. In addition to the protective effect of melanin against host defense in *A. fumigatus*, melanin is also a necessary component for conidia cell wall integrity. Moreover, *alb1* deletion in *A. fumigatus* revealed a surge in the fibronectin binding capacity and a reduction in laminin binding of the conidia [29]. Similar to *A. fumigatus*, the attenuation in virulence of *P. marneffei* in melanin-KD mutant could be due to a reduction of its adherence ability on the cell membrane of pulmonary epithelial cells during infection. The virulent properties of melanin in other pathogenic fungi have also been demonstrated using animal models. In *C. neoformans*, it has been shown that the genes involved in melanization also contributed to its dissemination from the lungs to other organs and even death of the host [23,45]. In *P. brasiliensis*, experimental infection with melanized fungal cells has led to higher fungal burdens in the animals when compared with non-melanized cells [25]. Furthermore, during infection, the expression of melanin synthesis genes was found to be increased in *P. brasiliensis* [46]. Similarly, for *S. schenckii* infection, melanized fungi showed a higher degree of dissemination in a mouse footpad model as compared to non-melanized mutants [47]. Fungal melanins can modulate host immune response. Melanized *P. brasiliensis* yeast cells were found to have increased resistance to phagocytosis [24]. This might be due to the fact that melanins are polymers that carry electric charges and their presence in the cell wall can alter the surface charge of the fungal cells and hence contribute to inhibition of phagocytosis [48]. On top of the reduction of phagocytosis, melanins can protect *P. brasiliensis* from macrophage killing [24]. A similar protective effect was also shown in *C. neoformans*, *Exophiala* species, *F*, *pedrosoi* and *S. schenckii* [31,49,50,51]. Improved survival of melanized cells is attributed to the protection of fungal cells against damages mediated by oxygen- or nitrogen-derived free radicals during phygocytosis [52]. Melanization also increases fungal resistance against killing by hypochlorite and hydrogen peroxide [53]. Melanins are effective scavengers of free radicals and they possess electron-transfer property [54]. In addition, in *A. fumigatus*, melanin can suppress apoptosis of macrophages that have already phagocytosed fungal conidia [55]. Melanin can change immune function by other means as well. In experimental mouse infections, melanin of *Cryptococcus* could modulate cytokine levels and triggers the complement system in response to infection [56,57]. Conversely, melanin of *A. fumigatus* suppressed the production of host cytokines, possibly by the blockage of pathogen-associated molecular pattern recognition by the immune system [44].

### 3.2. Yellow Pigment: Mitorubrinol and Mitorubrinic Acid

Mitorubrin, mitorubrinol and mitorubrinic acid are members of the azaphilone family, a structurally diverse group of natural products containing a highly oxygenated, bicyclic core and a chiral quaternary center [58]. The structure of mitorubrin and mitorubrinol were first elucidated from *P. rubrum* in 1965 [59]. Because of the capability of *P. rubrum* to produce high toxicity substances and the conspicuous yellow pigmentation, the colorants were suspected to be associated with livestock poisoning through feedstuffs that were infected by the fungus. However, bioassays showed that the yellow pigments, mitorubrin and mitorubrinol, in *P. rubrum* did not cause any toxicity in mice [59], implying that they were non-toxic compounds. The toxicity agent in *P. rubrum* was later confirmed to be rubratoxin B in 1968 [60,61]. After the discovery of mitorubrin and its derivatives, these pigments were gradually found as the main yellow colorants in the ascomata of *Talaromyces* species, including *T. austrocalifornicus*, *T. convolutes*, *T. emodensis*, *T. hachijoensis* and *T. wortmannii* var. *sublevisporus* [62], and *Penicillium* species, such as *P. funiculosum* [63]. Even though mitorubrin and its derivatives have been recognized as polyketides for decades, no *pks* genes or their biosynthetic pathways have been elucidated until this recent discovery in *P. marneffei*.

To search for the yellow pigment biosynthetic pathway in *P. marneffei*, all 25 *pks* genes in the *P. marneffei* genome were knocked down systematically [21]. A loss of yellow pigment was observed in the *pks11*-KD and *pks12*-KD mutants (Figure 2B–D). Ultra-high performance liquid chromatograph (UHPLC)-mass spectrometry (MS) and MS/MS analysis of the culture filtrates of wild type *P. marneffei* and the *pks11*-KD and *pks12*-KD mutants confirmed that the yellow pigment is composed of mitorubrinic acid and mitorubrinol [21]. Sequencing of the *pks11* and *pks12* genes revealed that they are in the same *pks* gene cluster involved in the biosynthesis of yellow pigment. *pks11*, containing an intron of 52 bp in length and encoding 2575 amino acid residues with an estimated molecular mass of 282.6 kDa, is 7780 bp in length; while *pks12*, containing an intron of 63 bp in size and encoding 1806 amino acid residues with an estimated molecular mass of 197.9 kDa, is 5485 bp in length. Both PKS11 and PKS12 belong to non-reducing PKSs [21]. During the production of mitorubrinol and mitorubrinic acid, PKS11 and PKS12 may probably function in a sequential manner [21]. PKS12 produces a tetraketide, orsellinic acid, which serves as an advanced precursor for PKS11. PKS11 possesses a putative starter unit-ACP transacylase (SAT) domain for processing the advanced starter unit to continue the extension process. In addition, PKS11 contains a methyltransferase domain which methylates the PKS products, using a methyl group donated by S-adenosylmethionine. Some polyketides, such as lovastatin and zearalenone, are also produced by two PKSs [64,65]. For instance, in *G. zeae*, the biosynthesis of zearalenone begins with PKS13, where a hexaketide, serving as a precocious starter unit for further processing by the second PKS, PKS4, is produced [65].

Functional studies of mitorubrinol and mitorubrinic KD mutants in *P. marneffei* (*pks11*-KD, *pks12*-KD and *pks11*, *pks12* double-KD mutants) showed a decrease in virulence *in vivo* (in a murine model) and *in vitro* (in mouse J774 macrophage and phorbol myristate acetate-induced human THP1 macrophage models) [21]. Given that mitorubrin is not poisonous in murine model [59], mitorubrinol and mitorubrinic acid might contribute to virulence by enhancing fungal survival inside macrophages. Moreover, the *pks11*, *pks12* double-KD mutants did not show an improved mouse/intracellular survival rate in both murine and macrophage models, indicating that the knockdown of either *pks11* or *pks12* was adequate to stop the biosynthesis of mitorubrinol and mitorubrinic acid [21]. This confirmed that PKS11 and PKS12 are sequentially involved in the biosynthesis of mitorubrinol and mitorubrinic acid [21].

### 3.3. Toxin and Red Pigment Biosynthetic Pathway: Monascorubrin, Rubropunctatin, Citrinin and Ankaflavin

*P. marneffei* produces diffusible red pigment when they grow at temperatures below 30 °C. Such a unique characteristic is important for the laboratory identification of this fungus. In 2007, Bhardwaj *et al.* purified the red pigment by reverse phase liquid chromatography and investigated the putative structure, which was claimed to have a structural resemblance with herquinone that contains a phenalene carbon framework and forms dimer through disulfide bond, using atomic absorption, ultra violet and visible fluorescence, infrared radiation, nuclear magnetic resonance and tandem mass spectrometry [66]. In 2009, in search for the fungal cell factories for the manufacture of natural food colorants, Mapari *et al.* reported that *P. marneffei* produces monascorubramine, a well-known red pigment polyketide produced by *Monascus* species [67]. In 2014, we systematically knocked down all 25 *pks* genes in *P. marneffei* and a complete loss of red pigment production was found in *pks3*-KD mutant (Figure 2E) [20]. By comparing the metabolomic profiles of the wild type and *pks3*-KD mutant using UHPLC-MS and MS/MS, the red pigment was revealed to be a mixture of more than 16 different chemical compounds, which are the amino acid conjugates of monascorubrin and rubropunctatin [20]. Furthermore, surprisingly, besides pigment production, PKS3 is also responsible for the production of a well-known toxin, citrinin, as well as a yellow pigment, ankaflavin [20]. The compound reported by Bhardwaj *et al.* was not observed in both the wild type strain and KD mutants of *P. marneffei* in their studies [20].

Current evidence at hand shows that the red pigment biosynthetic gene cluster in *P. marneffei* consists of five genes, which encode a polyketide synthase (PKS3), a transcription activator (RP1), a β-subunit fatty acid synthase (RP2), an α-subunit fatty acid synthase (RP3) and an oxidoreductase (RP4) [20]. The biosynthesis starts with the formation of a fatty acid chain (3-oxo-octanoic acid or 3-oxo-decanoic acid) from acetyl-CoA and malonyl-CoA, which is catalyzed by RP2, RP3 and RP4. RP1 is responsible for PKS3 activation. A pentaketide compound, citrinin, is synthesized by PKS3 using one acetyl-CoA and four malonyl-CoA [20]. By adding one more malonyl-CoA, a hexaketide compound, which is named compound **1** and is a diffusible orange pigment, is formed [20]. The newly synthesized fatty acid, 3-oxo-octanoic acid or 3-oxo-decanoic acid, is taken up by PKS3. PKS3 esterifies the fatty acid and compound **1** to produce two other orange pigments, monascorubrin or rubropunctatin, respectively. Monascorubrin or rubropunctatin, which possesses unique structural configurations with high affinity to primary amino compounds, is secreted outside the cell and conjugated with different amino acid residues in the culture media, forming red-colored compounds. Monascorubrin or rubropunctatin could also be reduced to give monacin or ankaflavin, respectively. *pks3*-KD and *rp1*-KD mutants showed an accumulation of 3-oxo-decanoic acid and a depletion of ankaflavin, citrinin, compound **1**, and monascorubrin [20]. No diffusible pigment was observed in both mutants. *rp2*-KD, *rp3*-KD and *rp4*-KD mutants showed a normal production of citrinin, an accumulation of compound **1** and a depletion of ankaflavin, monascorubrin and 3-oxo-decanoic acid [20]. The diffusible orange pigment, compound **1**, was observed in *rp2*-, *rp3*- and *rp4*-KD mutants. Owing to the structural resemblance between ankaflavin and monascorubrin, and given that ankaflavin could not be detected in *pks3*-, *rp1*-, *rp2*-, *rp3*- and *rp4*-KD mutants, ankaflavin is probably the reduced product of monascorubrin in the red pigment biosynthetic pathway [20].

Monascorubrin, rubropunctatin, and other structurally related compounds are well-known red pigments produced by *Monascus* species [68]. These natural pigments have been exploited as natural food colorants in the production of traditional oriental foods such as meats, vinegar, and bean curd [26]. Nowadays, these red pigments were also utilized in Western countries for coloring sausages and hams [69]. Similar to *P. marneffei*, *Monascus* species also excrete citrinin which possesses nephrotoxic and hepatotoxic properties [70]. Contamination of these toxic red pigments has raised a great concern in the food industry for the use of *Monascus* pigments as substitutes. Although *M. purpureus* and *P. marneffei* both produce monascorubrin and citrinin, *P. marneffei* uses one PKS cluster to synthesize both polyketides, whereas in *M. purpureus*, the biosynthesis are separated into two independent pathways. In 2005, Shimizu *et al.* discovered that a *pks* gene, *pksCT*, is necessary for citrinin production in *M. purpureus*. Gene disruption of *pksCT* leads to a total loss of citrinin biosynthesis but no substantial changes in the pigment production [71]. Furthermore, a transcription factor, *ctnA*, was also found upstream of *pksCT* and disruption of *ctnA* led to an extremely low citrinin biosynthesis [72]. Balakrishnan *et al.* also reported that the red pigment production in *M. purpureus* involves a *pks* gene and its potential transcriptional activators, MpPKS5 and mppR1 [73]. Gene deletion of *mppR1* caused a loss of monascorubrin and red pigment [73].

## 4. Concluding Remarks

The availability of genome sequences has revolutionized the research in many pathogenic microbes. *P. marneffei* is no exception. Genome sequencing and post-genomic studies have not only led to a better understanding of its phylogeny, fundamental biological properties and virulence determinants, but have also provided us with the tools for genotyping this important pathogenic thermally dimorphic fungus in Southeast Asia. The discovery of 25 *pks* genes in the *P. marneffei* genome has led to even more unanswered questions. Although four of the 25 genes have been shown in recent years to be responsible for the synthesis of various pigments in *P. marneffei*, the question remains as to what secondary metabolites the other 21 *pks* genes synthesize. How many of them are related to virulence, thermal dimorphism, antimicrobial properties, *etc.*? Hopefully, the parallel advancement of other disciplines, such as transcriptomics and metabolomics, will help provide answers to these important questions in the near future.
